# Vitamin D replacement therapy may regulate sleep habits in patients with restless leg syndrome

**DOI:** 10.1515/med-2024-1125

**Published:** 2025-02-11

**Authors:** Feyza Ustabaş Kahraman, Ayşegül Doğan Demir, Mebrure Yazıcı, Fatma Betül Çakır, Hayrettin Temel, Abdülhamit Çollak, Ufuk Erenberk

**Affiliations:** Child Health and Diseases, İstanbul Medipol University, İstanbul, Bağcilar, 34214, Turkey; Child Health and Diseases, Bezmialem Vakif University, İstanbul, Fatih, 34093, Turkey; Pediatric Hematology and Oncology, İstanbul Medeniyet University, İstanbul, Kadiköy, 34720, Turkey; Child Health and Diseases, İstanbul Medipol University, İstanbul, Bağcilar, 34214, Turkey; Children’s Chest Diseases, İstanbul University Cerrahpaşa, İstanbul, Fatih, 34098, Turkey; Child Health and Diseases, Bezmialem Vakif University, Topkapı, Adnan Menderes Blv., İstanbul, Fatih, 34093, Turkey

**Keywords:** child health and disease, restless leg syndrome, sleep habits, vitamin D

## Abstract

**Aim:**

The aim of this study is to determine the effect of vitamin D replacement therapy on sleep habits in restless legs syndrome (RLS) patients with vitamin D deficiency.

**Methods:**

The study was conducted between September 1, 2018, and September 1, 2019, at the Pediatrics outpatient clinic of Bezmialem Vakif University. 46 cases with RLS and 43 healthy controls between these dates were included in the study. The Children’s Sleep Habits Questionnare was filled out retrospectively by parents.

**Results:**

While vitamin D supplementation was given to 20 children with RLS who were found to have vitamin D deficiency, the 26 patients without vitamin D deficiency did not receive supplementation. There was no significant difference between the treatment groups in terms of gender, age, and pre-treatment sleep habits. Compared to the pre-treatment period, post-treatment bedtime resistance, sleep onset delay, sleep anxiety, parasomnias, daytime sleepiness, and CSHQ total score were significantly improved in RLS cases with vitamin D deficiency. There were no significant differences in RLS patients without vitamin D supplementation. Other comparisons also showed no difference between groups.

**Conclusion:**

There was no difference between the sleep habits of RLS cases with and without vitamin D deficiency. Sleep habits improved positively after vitamin D supplementation in patients with vitamin D deficiency compared to the pre-treatment period. There was no difference in the sleeping habits of RLS patients without vitamin D deficiency during the same period. Vitamin D supplementation can improve sleep habits in patients with RLS.

## 1 Introduction

Restless legs syndrome (RLS) is less common in children than in adults, but its effects on children can be more serious due to the essential role of sleep in their growth and development. The condition involves uncomfortable and distressing leg urges, especially during rest, which can greatly disrupt sleep quality and overall health [1]. It is believed that around 1 in every 50 children report experiencing symptoms associated with RLS at some point during their childhood [2].

The uncomfortable sensations associated with RLS make it challenging for patients to fall asleep, leading to poor sleep quality and daytime fatigue. Treatments aimed at reducing these sensations enable patients to develop healthier sleep patterns. Previous studies have demonstrated that vitamin D supplementation, a key treatment option for RLS, significantly alleviates pain severity in affected individuals [3,4,5,6]. Vitamin D levels are typically low in individuals with RLS, and research has focused on the hypothesis that vitamin D deficiency may play a significant role in the etiopathogenesis of RLS [4,6,7]. It has been proposed that dysfunction in the dopaminergic system contributes to the pathogenesis of RLS [8], and vitamin D contributes to the activity of the dopaminergic system [9]. It also appears that vitamin D has a protective effect against glutathione reduction due to toxins in mesencephalic dopaminergic neurons [9].

While many studies have confirmed the role of vitamin D treatment in reducing resting impulses in RLS patients, research on its effects on sleep habits remains scarce. In RLS cases with vitamin D deficiency, supplementation may enhance sleep habits and indirectly improve quality of life by lessening the severity of the condition. In this study, it was aimed to determine the effect of vitamin D replacement therapy on sleep habits in RLS patients with vitamin D deficiency. In addition, the sleep habits of children with RLS were compared with healthy controls.

## 2 Materials and methods

The study took place from September 1, 2018, to September 1, 2019, at the Pediatrics outpatient clinic of Bezmialem Vakif University. All cases diagnosed with RLS during this period were included.

Children aged 8–17 years, diagnosed with RLS and free of any other chronic conditions (e.g., diabetes mellitus, chronic kidney disease, rheumatological diseases, musculoskeletal disorders, and metabolic diseases), and not on any medication, were included in the study. For the control group, children who visited the Pediatric Outpatient Clinic at Bezmialem Vakif University Faculty of Medicine Hospital during the study period and had no underlying diseases were selected in a 1:1 ratio, age-matched with the RLS cases. Intermittent RLS is defined as restless leg symptoms that are bothersome enough to require treatment but occur less than twice a week on average. Chronic persistent RLS was defined as moderate or severe symptoms that are bothersome enough to require daily treatment and occur at least twice a week or more.

Vitamin D levels in RLS cases were assessed both before and after treatment. The cases were divided into two subgroups as vitamin D level below and above 30 ng/dl, and statistics were made by comparing these two groups. Additionally, all participants, including the control and RLS groups, completed the Children’s Sleep Habits Questionnaire (CSHQ) at the time of admission with parents filling out the questionnaire on behalf of the children. Both RLS groups also completed the CSHQ 3 months later.

### 2.1 Vitamin D supplementation

The RLS patients with vitamin D deficiency were prescribed 2000 IU oral vitamin D treatment for 6 weeks. Subsequently, 600–1,000 IU/ml of vitamin D support was provided to keep the blood levels of 25-OH vitamin D3 above 30 ng/ml. Patients without vitamin D deficiency were followed up without vitamin D supplementation.

### 2.2 CSHQ

The CSHQ, developed to investigate children’s sleep habits and sleep-related problems, consists of 33 items in total. Eight subscales are defined in the scale and can be listed as follows: bedtime resistance, sleep onset delay, sleep duration, sleep anxiety, night waking, parasomnia, sleep-disordered breathing, and daytime sleepiness [10]. The questionnaire was filled out retrospectively by the parents. Parents were asked to evaluate the sleeping habits of the child within the previous week.

### 2.3 Statistical analysis

All analyses were performed on SPSS v21 (SPSS Inc., Chicago, IL, USA). For the normality check, the Shapiro–Wilk test was used. Data are presented as mean ± standard deviation or median (25th–75th percentile) for continuous variables according to normality of distribution and as frequency (percentage) for categorical variables. Normally distributed variables were analyzed with two-way repeated measures analysis of variances (ANOVA). Non-normally distributed variables were analyzed with the Wilcoxon Signed Ranks test for repeated measurements. Directional relationships between variables were analyzed by calculating Spearman correlation coefficients. Comparison of the groups concerning these variables was performed by analyzing differences between the measurements with the Mann–Whitney U test or Student’s *t*-test. *p*-values <0.05 were accepted as statistically significant.


**Ethical approval:** Ethics committee approval was granted by the Bezmialem Vakif University Clinical Research Ethics Committee (Approval no: 16/13, date: 15/08/2018).
**Informed consent:** The participants in the study provided their written informed consent by signing the informed consent form prior to their inclusion in the study.

## 3 Results

In terms of gender, 30.4% of the RLS cases and 37.2% of the control group were male. The mean age was 9.7 ± 3.9 years in RLS cases and 10.1 ± 3.6 years in the control group. There was no significant difference between the groups in terms of gender and age (*p* = 0.499 and *p* = 0.452, respectively). In addition, there was no significant difference between the groups in terms of sleeping habits (*p* > 0.05). The summary of CSHQ scores with regard to groups is shown in Table 1.

**Table 1:** Summary of scores of CSHQ with regard to groups

	Groups		
	RLS (*n* = 46)	Healthy control (*n* = 43)	*p*
Bedtime resistance	8 (7–11)	8 (6.5–10)	0.332
Sleep onset delay	1 (1–2)	1 (1–1)	0.381
Sleep duration	3 (3–4)	3 (3–3.5)	0.582
Sleep anxiety	6 (4–8)	5 (4–8)	0.432
Night wakings	4 (3–5)	4 (3–5)	0.738
Parasomnias	9 (8–11)	8 (7–10)	0.242
Sleep-disordered breathing	3 (3–4)	3 (3–3.5)	0.256
Daytime sleepiness	12.5 (12–15)	8 (6.5–10)	0.587
Total score	52.20 ± 8.41	48.30 ± 5.20	0,061
Total daily sleep duration	9 (8–10)	9 (8–10.5)	0.278

Data are presented as the mean ± standard deviation or median (1st–3rd quartile) according to normality of distribution.

While vitamin D supplementation was given to 20 children with RLS who were found to have vitamin D deficiency, 26 patients without vitamin D deficiency were not given supplementation. Moreover, 34.6% of those who received vitamin D treatment and 25% of those who did not receive treatment were male. With regard to the characteristics of the subgroups in RLS patients, we found that the mean age was 9.8 ± 3.7 years in vitamin D recipients and 9.6 ± 4.2 in the group not receiving treatment. There was no significant difference between the RLS subgroups in terms of gender and age (*p* = 0.482 and *p* = 0.372, respectively). The subgroups’ pre-treatment sleep habits were also similar (*p* > 0.05). Compared to the pre-treatment period, post-treatment bedtime resistance, sleep onset delay, sleep anxiety, parasomnias, daytime sleepiness, and CSHQ total score were statistically significantly improved in RLS cases with vitamin D deficiency (*p* < 0.05). While the delay in sleep onset was significantly improved in the RLS group with vitamin D replacement (*p* = 0.034), there was no difference in sleep duration (*p* = 0.433). In the RLS group without vitamin D replacement, there was no significant difference in sleep onset delay and sleep duration at the 3-month follow-up.

We also examined correlations between vitamin D levels and CSHQ scores. Before treatment, there was no significant directional relationship between vitamin D level and disease severity (*r* = −0.115, *p* = 0.577). For the post-treatment analysis, we only included patients who had received vitamin D treatment. The results again showed no significant correlation between vitamin D and the severity of symptoms (*r* = −0.028, *p* = 0.891).

There was no significant difference in the other scores and the RLS cases without vitamin D supplementation. A summary of CSHQ scores of RLS patients with regard to treatment subgroups is shown in Table 2.

**Table 2:** Summary of scores of RLS patients CSHQ with regard to treatment

	Groups in RLS						
	Vitamin D treatment (*n* = 20)			Without treatment (*n* = 26)			
	Pre-treatment	Post-treatment	*p*	Pre-treatment	Post-treatment	*p*	p*
Bedtime resistance	8 (6–11)	7 (6–8)	**0.010**	8.5 (7–10)	6.5 (6–7)	0.273	0.670
Sleep onset delay	1 (1–2)	1 (1–1)	**0.034**	1 (1–2)	1 (1–1)	0.180	0.866
Sleep duration	3 (3–4)	3 (3–3)	0.433	3 (3–5)	3 (3–5)	1.00	0.496
Sleep anxiety	6 (4–8)	4 (4–5)	**0.009**	6.5 (5.5–9.5)	4 (4–5)	0.109	0.091
Night wakings	5 (3–5)	5 (3–5)	0.926	3.5 (3–5)	3.5 (3–5)	0.257	0.332
Parasomnias	9 (8–11)	7 (7–9)	**0.002**	9 (7–11.5)	7.5 (7–9)	0.109	0.875
Sleep-disordered breathing	3 (3–4)	3 (3–3)	0.107	3 (3–4)	3 (3–3)	0.317	0.312
Daytime sleepiness	14 (12–16)	12 (10–15)	**0.006**	12 (12–13.5)	12 (10–15)	0.357	0.164
Total	54 (46–60)	46 (41–50)	**<0.001**	50 (46–56.5)	43 (37–52)	0.072	0.919
Total daily sleep duration	9 (8–9.5)	9 (8.5–9)	0.115	9 (8–10)	9 (9–10)	0.414	0.650

Data are presented as median (1st–3rd quartile). *Comparison of initial values (pre-treatment) between groups.

All results were considered significant if the *p* value was less than 0.05 at 95% CI.

## 4 Discussion

Vitamin D, a crucial fat-soluble vitamin, plays a key role in various bodily functions, particularly in bone metabolism. However, its deficiency can cause a broad spectrum of symptoms across different systems and organs. In this study, which aimed to assess the impact of vitamin D replacement therapy on sleep habits in RLS patients with vitamin D deficiency, significant improvements were observed in areas such as bedtime resistance, sleep onset delay, sleep anxiety, parasomnias, and daytime sleepiness following the therapy. The comparison of results suggests an impact of vitamin D in improvements; however, the absence of directional relationships in post hoc analyses does not suggest a measurable effect. This may be associated with numerous factors and is possibly due to the indirect relationships between vitamin D levels and RLS.

In RLS cases, vitamin D helps reduce unpleasant urges, regulate sleep habits, and promote adequate sleep. A meta-analysis by Gao et al. on vitamin D deficiency and sleep issues found that individuals with vitamin D deficiency face a 1.5-fold higher risk of developing sleep problems [11]. Numerous studies have investigated the relationship between vitamin D deficiency and various sleep characteristics, consistently showing that low vitamin D levels negatively affect different aspects of sleep and generally lower overall sleep quality [12,13,14,15]. In a study assessing the impact of vitamin D levels on sleep habits in children with autism spectrum disorder, Guler et al. demonstrated that all subscales of the CSHQ, as well as the total score, improved following vitamin D treatment [16]. In our study, consistent with the literature, vitamin D supplementation was found to have positive effects on nearly all dimensions of sleep habits. Although various aspects of vitamin D physiology have been extensively studied, its exact mechanism of action on sleep remains unclear. Both human and animal studies suggest that vitamin D may influence sleep-related regions in the central nervous system, thus contributing to sleep regulation and characteristics [17,18]. Vitamin D deficiency also appears to increase myopathic pain, similar to that experienced in RLS and negatively impacts sleep quality [19].

Vitamin D reduces sleep onset delay by easing unpleasant urges in RLS cases. Majid et al. found that vitamin D supplementation lowered sleep latency in adults with sleep problems [20]. In a similar study by Huang et al., vitamin D deficiency was linked to the development of sleep onset delay, and supplementation was found to positively impact sleep latency [21], which has been reported before, as demonstrated by reduced sleep latency with vitamin D supplementation [22]. We found that sleep onset delay decreased in RLS patients receiving vitamin D supplementation, consistent with prior literature. Additionally, our study showed that sleep anxiety and bedtime resistance improved with vitamin D supplementation. Sleep habits and sleep problems are closely interconnected [18]. The vitamin D-receiving RLS group experienced a significant improvement in sleep onset delay, bedtime resistance, sleep anxiety, parasomnia, and daytime sleepiness at the 3rd-month follow-up. It was, therefore thought that these sleep disorder symptoms could be associated with vitamin D levels, indicating bilateral relationships among these factors. This hypothesis is supported by Gao et al.’s study, which demonstrated that vitamin D deficiency negatively impacted sleep habits and suggested direct relationships between the factors contributing to sleep disturbances in the context of reduced vitamin D levels [11].

Negative sleep habits can lead to increased daytime sleepiness, a greater need for naps, and reduced work efficiency, potentially resulting in accidents, health issues, and workforce loss. In Gao et al.’s meta-analysis, it was shown that vitamin D deficiency raised the risk of daytime sleepiness by 1.36-fold [11]. This has been supported by other research showing a significant relationship between vitamin D deficiency and daytime sleepiness [23]. In different case–control studies [24,25] and cross-sectional studies [26], vitamin D deficiency has been shown to cause daytime sleepiness (through the examination of the relationships between vitamin D levels and sleep habits). In our study, consistent with prior research, it was shown that daytime sleepiness levels decreased among subjects with RLS who received vitamin D supplementation due to deficiency. In addition, another sleep habit evaluated in our study, parasomnia, was determined to be mitigated by vitamin D supplementation. Results consistent with our study have been published in the literature. For instance, Ozkaya and Gungor showed lower vitamin D levels in children with sleep terror compared to healthy children [27]. Vitamin D supplementation was thought to be effective in reducing parasomnia complaints both directly and indirectly due to the improvement in other sleep habits.

One of the important limitations of our study is that the post-treatment vitamin D levels of RLS patients were not examined. Although vitamin D supplementation was provided, changes in serum vitamin D levels in both groups were not evaluated. In previous studies, it has been shown that both vitamin D supplementation and placebo reduce RLS severity [28]. The placebo effect was not taken into account in our study; that is, patients who received vitamin D supplements and those who did not were aware of their treatment status. In both groups, factors that may positively affect vitamin D levels other than vitamin D supplementation, such as light exposure and nutrition, were not investigated. The fact that sleep habits and various factors that may affect RLS severity were not examined in our study may also reduce the attribution of the results to vitamin D supplementation.

## 5 Conclusion

There was no difference between the sleep habits of RLS cases without vitamin D deficiency and RLS cases with vitamin D deficiency. Furthermore, while sleep habits improved positively after vitamin D supplementation in patients with vitamin D deficiency (compared to the pre-treatment period), there was no difference in the sleeping habits of RLS patients without vitamin D deficiency during the same period. Vitamin D supplementation can improve sleep habits in patients with RLS.
